# H_2_S Donor Therapy Reverses Established Pulmonary Arterial Hypertension and Pulmonary Vascular Structural Remodeling in Rats

**DOI:** 10.3390/biomedicines14040760

**Published:** 2026-03-26

**Authors:** Jie Zheng, Yanan Zhang, Boyang Lv, Yuanyuan Ma, Xuecong Zhong, Junbao Du, Hongfang Jin, Yaqian Huang

**Affiliations:** 1 Department of Pediatrics, Peking University First Hospital, Beijing 100034, China; zhengjie22@bjmu.edu.cn (J.Z.); zhangyananfuli@163.com (Y.Z.); lvboyang@pku.edu.cn (B.L.); 15210394375@163.com (X.Z.); drjunbaodu@pku.edu.cn (J.D.); 2 Laboratory of Animal Facility, Peking University First Hospital, Beijing 100034, China; mayyuan168@sina.com; 3 State Key Laboratory of Vascular Homeostasis and Remodeling, Peking University, Beijing 100191, China

**Keywords:** hydrogen sulfide, therapy, pulmonary arterial hypertension, pulmonary artery smooth muscle cell, proliferation

## Abstract

**Objectives**: Downregulation of the endogenous gasotransmitter hydrogen sulfide (H_2_S) contributes to the pathogenesis of pulmonary arterial hypertension (PAH). While prophylactic H_2_S supplementation prevents PAH initiation in different rat models, its ability to reverse fully established PAH and pulmonary vascular structural remodeling is unknown. In this study, we aimed to test whether H_2_S donor therapy can reverse the existing PAH in a chronic-hypoxia rat model. **Methods**: After 3 weeks of hypoxia exposure, rats with established hypoxia-induced pulmonary hypertension (HPH) were randomized to receive either continued hypoxia alone or hypoxia plus the H_2_S donor NaHS (56 μmol/kg·d, ip) for an additional 6 weeks. Pulmonary artery pressure, pulmonary artery muscularization, and right ventricular hypertrophy were assessed. Furthermore, the cell proliferation (Ki-67 and PCNA), ERK1/2 phosphorylation, and persulfidation of the endothelin type A receptor (ETAR) were examined and detected in rat lung tissues and pulmonary artery smooth muscle cells (PASMCs). **Results**: H_2_S therapy effectively reversed established HPH and pulmonary artery structural remodeling, reducing RVSP, mPAP, and the proportion of fully muscularized small pulmonary arteries by 13.8%, 12.0%, and 62.7%, respectively. Moreover, the PAT/PET ratio was normalized to normoxic levels. The right ventricular hypertrophy index decreased by 29.2%. Mechanistically, H_2_S therapy suppressed PASMC proliferation, reduced ERK1/2 phosphorylation, and enhanced ETAR persulfidation. Furthermore, dithiothreitol-mediated reduction of ETAR persulfidation abrogated these antiproliferative effects of H_2_S therapy, establishing persulfidation as an obligatory mechanism. **Conclusions:** H_2_S donor therapy effectively reverses established HPH and pulmonary vascular structural remodeling by inhibiting PASMC proliferation, which is linked to enhanced ETAR persulfidation. These data provide preclinical proof-of-concept for H_2_S-based interventions in patients with manifest PAH.

## 1. Introduction

Persistent pulmonary vasoconstriction and multifactorial pulmonary vascular structural remodeling are the key pathophysiological features of pulmonary arterial hypertension (PAH). Together they drive sustained luminal narrowing and a progressive rise in pulmonary vascular resistance, ultimately leading to right ventricular hypertrophy and failure. The Global Burden of Disease Study 2021 reported an 85.6% increase in the prevalence of PAH over the past three decades [1,2] with an estimated global prevalence standing at approximately 1%. This upward trend underscores PAH as a substantial and rapidly growing global health burden [3]. Current therapeutic strategies target three canonical pathways: the prostacyclin pathway (prostacyclin analogs or prostacyclin receptor agonists), the nitric oxide pathway (phosphodiesterase-5 inhibitors or soluble guanylate cyclase stimulators), and the endothelin pathway (endothelin receptor antagonists) [4,5,6]. Mono- or combination therapy within these pathways improves clinical symptoms and hemodynamic parameters, leading to an increase in the 5-year survival rate. However, their efficacy in reversing established pulmonary vascular structural remodeling remains limited [7]. Consequently, PAH still carries high morbidity and mortality, with a 5-year mortality rate still approaching 50% [8,9,10,11]. Therefore, the identification of novel therapeutic targets capable of durably ameliorating pulmonary vascular structural remodeling remains an urgent unmet need in cardiovascular medicine.

Hydrogen sulfide (H_2_S), the third endogenous gaseous signaling molecule, has emerged as a key regulator of pulmonary vascular homeostasis. It is generated primarily from homocysteine and L-cysteine by cystathionine-γ-lyase (CSE), cystathionine-β-synthase (CBS), and 3-mercaptopyruvate sulfurtransferase (MPST) and maintains tonic vasorelaxation under physiological conditions [12,13]. Clinical and experimental evidence converge on a single theme: H_2_S deficiency is an important driver of PAH. Children with congenital heart disease-associated PAH exhibit that circulating H_2_S and CSE levels inversely correlate with disease severity [14,15]. Identical reductions in CSE expression and H_2_S content are reproduced in every major rat model of PAH—hypoxia, monocrotaline, and high pulmonary blood flow—mirroring the human phenotype. Conversely, daily supplementation with sodium hydrosulfide (NaHS, an H_2_S donor) throughout model induction normalizes pulmonary artery pressure and attenuates pulmonary vascular structural remodeling [16,17,18]. Genetic proof-of-concept was provided by CSE- or CBS-null mice, which developed exaggerated pulmonary vascular structural remodeling compared with wild-type littermates [19,20]. Further mechanistic studies reveal that H_2_S suppresses PAH and pulmonary vascular structural remodeling through pleiotropic actions: direct pulmonary vasodilation [21,22,23], blockade of endothelial-to-mesenchymal transition [24], inhibition of pulmonary artery smooth muscle cell (PASMC) proliferation [19,25], induction of PASMC apoptosis [26], suppression of collagen deposition and elastin expression [27], and anti-inflammatory and antioxidative actions [28,29]. Collectively, these findings suggest that H_2_S concurrently modulates pulmonary artery pressure and vascular structural remodeling, highlighting its therapeutic promise for PAH.

To date, most preclinical studies have pursued prevention rather than cure: H_2_S donors are administered before or concomitantly with the injurious stimulus and continued throughout model induction. This paradigm sharply diverges from the clinic, where patients present only after pulmonary vascular structural remodeling and hemodynamic compromise are entrenched. Whether an established, remodeled pulmonary circulation can be rescued by H_2_S remains untested. Here, we posed a deliberately late-intervention question: if H_2_S therapy is initiated only after severe PAH is fully established, can it regress occlusive vascular lesions and restore normal hemodynamics? Answering this question would narrow the translational gap and provide the experimental platform required to advance H_2_S from laboratory curiosity to disease-reversing therapy.

## 2. Materials and Methods

### 2.1. Animal Model

Healthy male Sprague Dawley rats (6 weeks old, 180–220 g) were purchased from SPF (Beijing, China) Biotechnology Co., Ltd. All procedures were approved by the Animal Ethics Committee of Peking University First Hospital (Ethics No. J2024004) and complied with the National Institutes of Health Guide for the Care and Use of Laboratory Animals. After one week of acclimatization, the rats were randomly allocated to different groups. The randomization sequence was generated using a random number table. A total of 36 rats were used in this study. The individual rat was considered the experimental unit. In a 3-week hypoxia exposure experiment, rats were randomly allocated into two groups, normoxia and hypoxia, with six rats per group. In a 9-week hypoxia exposure experiment, rats were randomly allocated into four groups: normoxia, hypoxia, hypoxia + NaHS, and normoxia + NaHS, with six rats per group. The sample size was chosen based on common practice in the field for similar types of experiments, which typically uses 3–6 animals per group to achieve reliable and reproducible results. Rats in the hypoxia and hypoxia + NaHS groups were housed continuously in a normobaric hypoxic chamber flushed with 10% O_2_; silica gel and soda lime controlled humidity and carbon dioxide. Bedding, food, and water were replaced once weekly at a fixed time. Additionally, the same experimenter performed the procedure for all animals to minimize inter-operator variability. After 3 weeks of hypoxic exposure to establish hypoxia-induced pulmonary hypertension (HPH), rats in the normoxia + NaHS and hypoxia + NaHS groups received daily intraperitoneal NaHS (56 μmol/kg, 161527, Sigma-Aldrich, Burlington, MA, USA) [19,30], while rats in the normoxia and hypoxia groups received an equal volume of normal saline between 15:00 and 17:00 PM. Daily administration was chosen to maintain steady-state drug levels. All injections were performed in a dedicated procedure room adjacent to the housing facility. Treatments and respective oxygen exposures continued for a further 6 weeks, giving a total study duration of 9 weeks. If an animal died prematurely before reaching the experimental endpoint, it was excluded from this study.

### 2.2. Echocardiography

Echocardiography was performed with a Vevo 2100 ultrasound system (FUJIFILM VisualSonics Inc., Toronto, ON, Canada) under 2% isoflurane anesthesia. Echocardiographic procedures and quantitative measurements were performed and analyzed by a qualified technician who was blinded to the group allocations. Pulsed-wave Doppler spectra of the pulmonary artery were obtained from a modified parasternal long-axis view to record pulmonary ejection time (PET) and pulmonary acceleration time (PAT). The PAT/PET ratio, which inversely reflects right ventricular afterload, was calculated as a noninvasive index of pulmonary artery pressure [31]. Two data points of PAT/PET from one animal in the group normoxia and one animal in the group normoxia + NaHS were excluded due to the failure of the test caused by the unstable heart rate after anesthesia.

### 2.3. Measurement of Mean Pulmonary Artery Pressure (mPAP)

Rats were anesthetized with intraperitoneal 2.5% avertin solution (1.5 mL/100 g) and positioned supine. A midline cervical incision exposed the right external jugular vein; the distal end was ligated, and a loose ligature was placed proximally. A microvascular clamp temporarily occluded blood flow. A small V-shaped venotomy was created, and a pressure catheter was inserted and secured by tightening the proximal ligature. The catheter was advanced into the right ventricle (RV) to record right ventricular systolic pressure (RVSP), then guided by the pressure waveform into the pulmonary artery to obtain mPAP. Surgical catheterization and data analysis were performed by a blinded technician.

### 2.4. Assessment of Right Ventricular Hypertrophy Index (RVHI)

After euthanasia, the rat heart was excised, immediately rinsed in cold PBS, and blotted. Atria were trimmed away, and the free wall of the right ventricle (RV) was carefully dissected from the left ventricle (LV) and septum (S). Wet weights were recorded and right ventricular hypertrophy was expressed as RV/(LV + S) (RV hypertrophy index, RVHI).

### 2.5. Elastic Van Gieson (EVG) Staining

Rat lung tissues were fixed in 4% paraformaldehyde, dehydrated, paraffin-embedded, and sectioned at 3 μm. Slides were immersed in EVG solution to visualize elastic laminae, rinsed in tap water and mounted.

### 2.6. Vascular Wall Geometry

Pulmonary arteries (15–50 μm external diameter) were selected from EVG-stained sections. Relative media thickness (RMT) was calculated as the mean distance between the external and internal elastic laminae; relative media area (RMA) was calculated as the area between the external and internal elastic laminae. For each rat, 4–20 pulmonary arteries per section were imaged with an optical microscope and analyzed using Leica Qwin software (version Q550 CW). The measurement of vascular morphology data was performed by an author who was blinded to the group assignments.

### 2.7. Immunofluorescence Quantification

After heat-mediated antigen retrieval in citrate buffer (pH 6.0) and blocking, tissue sections were incubated overnight at 4 °C with rabbit anti-vWF (1:100, 27186-1-AP, Proteintech, Wuhan, China), mouse anti-α-SMA (1:50, ZM-0003, ZSGB-BIO, Beijing, China), and rabbit anti-Ki67 (1:50, ab15580, Abcam, Cambridge, MA, USA), followed by Alexa Fluor 555-conjugated goat anti-rabbit IgG (1:500, A-21428, ThermoFisher, Waltham, MA, USA) and Alexa Fluor 488-conjugated goat anti-mouse IgG (1:500, A-11001, ThermoFisher, USA). Pulmonary arterioles (15–50 μm external diameter) were imaged by confocal microscopy. Vascular muscularization was graded by α-SMA coverage: <25% = non-muscularized; 25–74% = partially muscularized; ≥75% = fully muscularized (FM). For each animal, the proportion of FM arterioles was calculated.

### 2.8. H_2_S Content Measurement

The levels of H_2_S in rat plasma and lung tissues were measured as previously described [32]. Briefly, a selective H_2_S sensor was equilibrated in 0.05 M PBS to achieve a stable baseline current and then calibrated. Calibration was performed by immersing the sensor tip (~10 mm) into a series of Na_2_S standard solutions at concentrations of 0.5, 1, 4, 8, 16, and 32 μM. A calibration curve relating H_2_S concentration (µM) to sensor output signal was established. Subsequently, the sensor tip was immersed into the sample solution, and the output signal was recorded. At last, the H_2_S content in the sample was calculated using the established calibration curve.

### 2.9. Human PASMCs (hPASMCs) Culture and Treatment

The hPASMCs (Procell, Wuhan, China) were cultured in specialized medium at 37 °C and 5% CO_2_. After overnight starvation in serum-free conditions, hPASMCs in the hypoxia group were transferred to a hypoxic incubator (1% O_2_, 5% CO_2_, 37 °C) for 12 h. Subsequently, 100 µM NaHS or 100 µM GYY4137 [18] (HY-107632, MCE, Shanghai, China) was added, with or without 50 µM dithiothreitol (DTT), and incubation continued for a further 24 h under the same hypoxic conditions. The culture medium containing 100 μM NaHS was refreshed every 12 h during the experiment. Control cells were maintained under normoxic conditions throughout the entire experimental period.

### 2.10. Immunoblotting

Rat lungs and cell lysates were prepared in lysis buffer supplemented with protease inhibitors. Total proteins were separated by sodium dodecyl sulfate–polyacrylamide gel electrophoresis and transferred onto nitrocellulose membranes. After blocking with 5% non-fat milk, membranes were incubated overnight at 4 °C with the following primary antibodies: rabbit anti-CSE (1:1000, 12217-1-AP, Proteintech, China), mouse anti-CBS (1:2000, 67861-1-Ig, Proteintech, China), mouse anti-MPST (1:1000, sc-376168, Santa Cruz, Dallas, TX, USA), mouse anti-PCNA (1:5000, 60097-1-Ig, Proteintech, China), rabbit anti-Phospho-ERK1/2 (1:1000, AF1891, Beyotime, Shanghai, China), rabbit anti-ERK1/2 (1:1000, AF1051, Beyotime, China), rabbit anti-endothelin type A receptor (1:1000, ab178454, Abcam, USA), rabbit anti-β-actin (1:5000, 20536-1-AP, Proteintech, China), and mouse anti-β-tubulin (1:1000, TA-10, ZSBIO, China). Membranes were then probed with appropriate secondary antibodies for 1 h at room temperature and visualized using a horseradish peroxidase-labeled chemiluminescent detection reagent.

### 2.11. Detection of Protein Persulfidation via Biotin Switch Assay (BSA)

ETAR persulfidation was quantified by the BSA as previously described [19]. In brief, supernatants from rat lung tissue or cell lysates were collected and mixed with a blocking buffer containing 2.5% SDS and 20 mM methyl methanethiosulfonate. The mixture was incubated at 50 °C with shaking for 20 min. Subsequently, cold acetone was added, and the mixture was incubated at −20 °C for 2 h to remove excess methyl methanethiosulfonate. Protein precipitates were collected by centrifugation at 5000× *g* for 10 min at 4 °C and resuspended in 200 μL of lysis buffer. EZ-Link™ iodoacetyl-PEG2-Biotin (6 μL) was added and incubated overnight at 4 °C with gentle shaking. Biotinylated proteins were precipitated using NeutrAvidin UltraLink Resin^TM^ at 4 °C for 4 h. Finally, bead-bound persulfidated proteins were collected for subsequent immunoblotting analysis.

### 2.12. Statistical Analysis

Data were presented as mean ± standard error of the mean (SEM). Statistical analyses were performed using GraphPad Prism software (version 10.4.1). Differences between the two groups were determined by an unpaired Student’s *t*-test. Comparisons among multiple groups were conducted using one-way ANOVA followed by Tukey’s post hoc test. Normality and homogeneity of variances were assessed using the Shapiro–Wilk test and the Brown–Forsythe test, respectively. After testing, the data were confirmed to be normally distributed, and the variances were homogeneous across groups. *p* < 0.05 was considered statistically significant.

## 3. Results

### 3.1. H_2_S Treatment Alleviates Established Hypoxic HPH and Right Ventricular Hypertrophy in Rats

Three weeks of continuous hypoxia exposure on rats produced the expected HPH phenotype: mPAP and RVSP doubled and the PAT/PET ratio fell by 21.1%, as well as RVHI, RMT and RMA rose by 51.5%, 57.6%, and 49.6% relative to normoxic controls (Figure S1A–G). These data validated the model and provided a pathological baseline for late-intervention studies.

**Figure 1:**
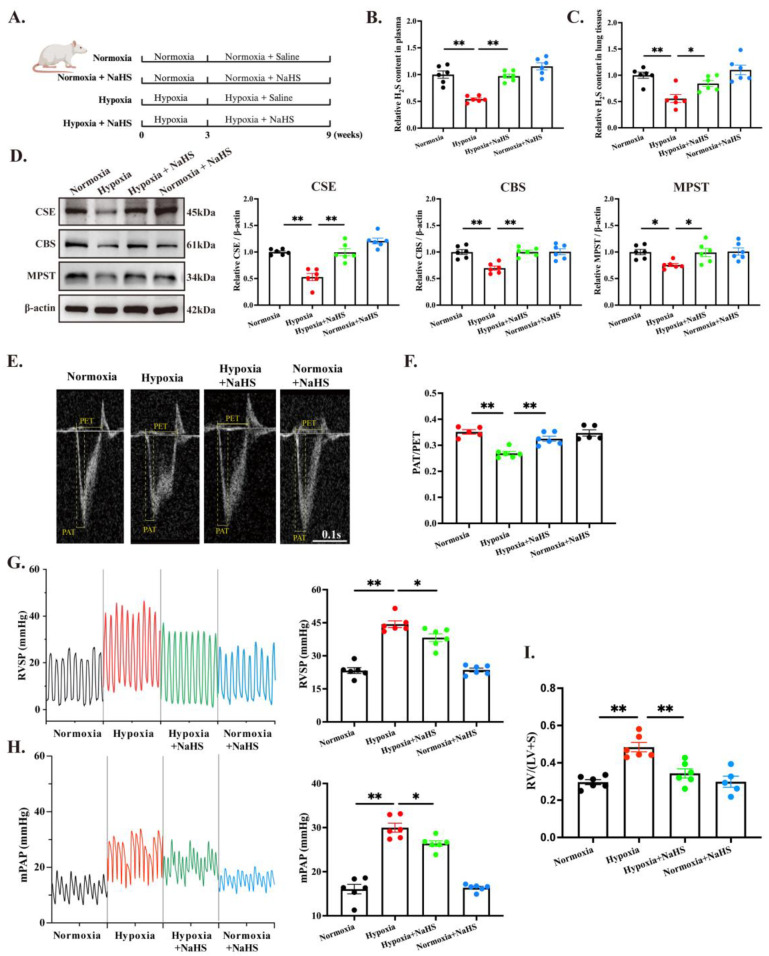
H_2_S treatment attenuated HPH and right ventricular hypertrophy in rats. (**A**). Schematic diagram of animal grouping and experimental procedures. (**B**). Plasma H_2_S levels measured by the sensitive sulfur electrode method. (**C**). Lung tissue H_2_S levels measured by the sensitive sulfur electrode method. (**D**). Protein expression of CSE, CBS, and MPST in lung tissue detected and quantified by immunoblotting. (**E**). Representative images of pulmonary arterial blood flow spectra obtained by echocardiography, scale bar = 0.1 s. (**F**). Statistical analysis of the echocardiographic index PAT/PET ratio. (**G**). RVSP measured by right heart catheterization. (**H**). mPAP measured by right heart catheterization. (**I**). Statistical analysis of the RVHI. All data are presented as mean ± SEM, *n* = 5–6. * *p* < 0.05, ** *p* < 0.01.

Subsequently, rats were treated with the H_2_S donor NaHS for 6 weeks under either normoxic or hypoxic conditions throughout the treatment period. The animal grouping and experimental design are illustrated in the flowchart (Figure 1A). Compared with the normoxia group, the hypoxia group exhibited significantly lower plasma H_2_S levels (Figure 1B), reduced H_2_S content in lung tissue (Figure 1C), and decreased protein expression of CSE, CBS, and MPST in the lung (Figure 1D). H_2_S supplementation restored both circulating and pulmonary H_2_S levels and effectively reversed the hypoxia-induced downregulation of CSE, CBS, and MPST in the lung tissue (Figure 1B–D).

Noninvasive echocardiography showed that NaHS treatment restored the PAT/PET ratio in hypoxic rats (0.33 ±0.01 vs. 0.27 ± 0.01), indicating a marked reduction in pulmonary artery pressure (Figure 1E,F). Right heart catheterization showed that NaHS treatment significantly lowered RVSP from 44.36 ± 1.53 to 38.24 ± 1.76 mmHg (a 13.8% decrease) and mPAP from 29.98 ± 1.02 to 26.38 ± 0.64 mmHg (a 12.0% decrease). These findings confirm that H_2_S supplementation effectively reverses established HPH and ameliorates hemodynamics dysfunction, an outcome that aligns with the echocardiographic data. Concomitantly, RVHI decreased from 0.48 ± 0.03 to 0.34 ± 0.02 (a 29.2% decrease, Figure 1I), demonstrating the regression of established right ventricular hypertrophy.

Collectively, late-initiated H_2_S donor therapy lowers pulmonary pressure and reverses maladaptive right ventricular remodeling in HPH.

### 3.2. H_2_S Treatment Reversed Established Pulmonary Vascular Structural Remodeling in HPH Rats

**Figure 2:**
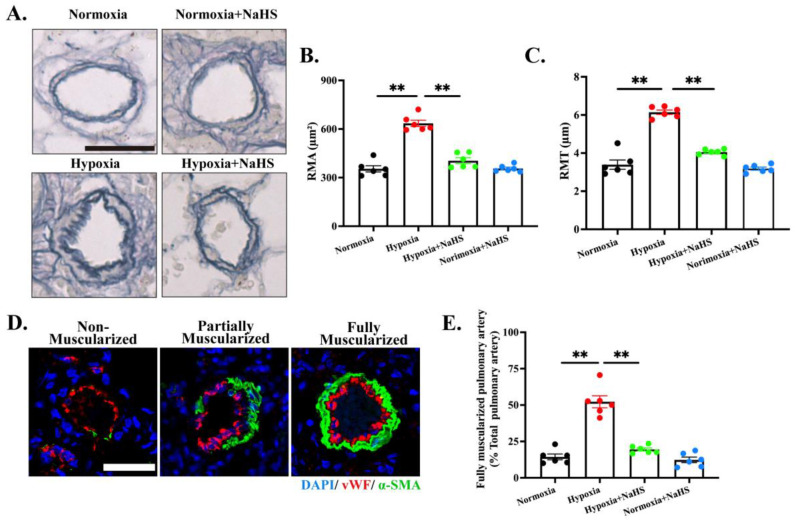
H_2_S treatment reversed pulmonary vascular structural remodeling in HPH rats. (**A**). Representative image of rat lung tissue stained with EVG, scale bar = 30 μm. (**B**,**C**). Statistical analysis of RMA and RMT of small pulmonary arteries. (**D**). Representative images showing non-muscularized, partially muscularized, and fully muscularized vessels in lung tissue; scale bar = 50 μm. (**E**). Statistical analysis of the percentage of fully muscularized vessels in rat lung tissue. All data are presented as mean ± SEM, *n* = 6. ** *p* < 0.01.

To evaluate the effect of H_2_S treatment on pulmonary vascular structure, we combined histomorphometry with molecular biological analyses. Histomorphometry of EVG-stained sections showed that hypoxia doubled both RMA and RMT in small pulmonary arteries. NaHS restored both parameters to normoxic values, preserving luminal patency (Figure 2A–C). Confocal analysis of α-SMA revealed that hypoxia nearly quadrupled the proportion of fully muscularized small pulmonary arteries (14.4% to 52.3%), while late-initiated H_2_S donor therapy reversed this shift, reducing the fully muscularized fraction to 19.5%—indistinguishable from normoxic controls (Figure 2D,E). Thus, H_2_S not only lowers pressure but also regresses established medial hypertrophy and pathological muscularization in hypoxic HPH.

### 3.3. H_2_S Treatment Silenced the ERK1/2 Pathway and Inhibited PASMC Proliferation in HPH Rats

**Figure 3:**
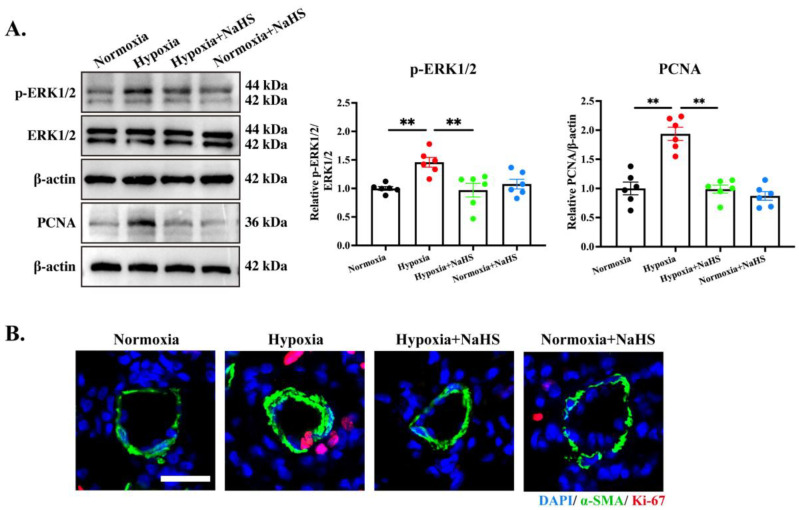
H_2_S treatment inhibited PASMC proliferation in HPH rats. (**A**). Western blot detection and quantitative analysis of p-ERK1/2 levels and PCNA protein expression in rat lung tissue. (**B**). Immunofluorescence micrographs of small pulmonary vessels co-stained for α-SMA (green), Ki-67 (red), and DAPI (blue); scale bar, 50 μm. All data are presented as mean ± SEM, *n* = 6. ** *p* < 0.01.

To investigate the potential mechanisms by which H_2_S therapy reverses HPH and pulmonary vascular structural remodeling, we examined changes in key proliferative signaling molecules. Hypoxia doubled the phospho-ERK1/2/total-ERK1/2 ratio and PCNA expression in lung tissues of rats (Figure 3A). Confocal imaging revealed a robust increase in Ki-67^+^ PASMCs (Figure 3B). NaHS reversed each of these changes, restoring phospho-ERK1/2, PCNA and Ki-67 to normoxic levels (Figure 3A,B). Thus, late-initiated H_2_S donor therapy curbs pathological PASMC proliferation by disabling the ERK1/2 proliferative axis, which might underlie its regression of HPH and pulmonary artery structural remodeling.

### 3.4. H_2_S Inhibits PASMC Proliferation and Reverses Pulmonary Vascular Structural Remodeling by Persulfidating ETAR

**Figure 4:**
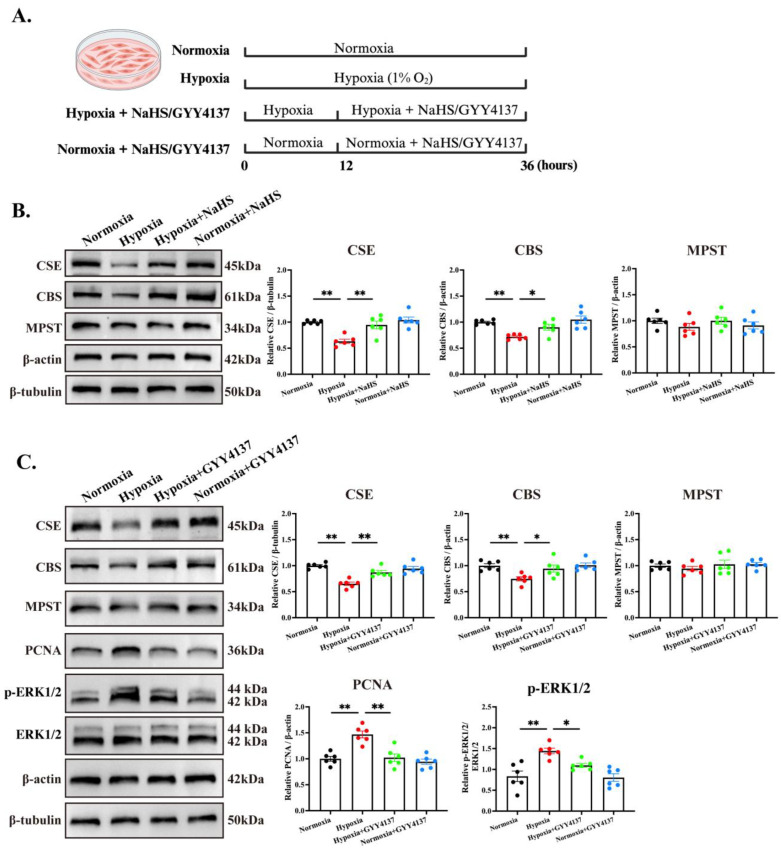
H_2_S donors restored the expression of key H_2_S-producing enzymes and antagonized hypoxia-induced proliferation of hPASMCs. (**A**). Schematic diagram of cell grouping and treatment regimen. (**B**). Immunoblotting assays and quantitative analysis showing the effects of hypoxia exposure and NaHS treatment on the protein expression levels of CSE, CBS, and MPST in hPASMCs. (**C**). Immunoblotting assays and quantitative analysis showing the effects of hypoxia exposure and GYY4137 treatment on the protein expression levels of CSE, CBS, MPST, PCNA, and phospho-ERK1/2 in hPASMCs. All data are presented as mean ± SEM, *n* = 6. * *p* < 0.05, ** *p* < 0.01.

To further elucidate the potential mechanism by which H_2_S exerts therapeutic effects in HPH, we first evaluated the effects of hypoxic exposure and NaHS treatment on the key enzymes responsible for H_2_S production and intracellular H_2_S levels at the cellular level. Following a 12 h incubation of hPASMCs under 1% O_2_ conditions, a marked downregulation of CSE expression was observed (Figure S2). Based on this model, NaHS was subsequently administered for 24 h prior to further detections (Figure 4A). The fluorescent probe assays confirmed that after 24 h of NaHS supplementation, the intracellular H_2_S levels were significantly increased (Figure S3). Furthermore, compared with the normoxic group, hypoxia exposure significantly reduced the protein expression levels of CSE and CBS in hPASMCs to 63.2% and 71.7% of the normoxic control group, respectively. Notably, NaHS treatment effectively reversed the hypoxia-induced downregulation of CSE and CBS (Figure 4B). In addition, neither hypoxia exposure nor NaHS treatment had a significant effect on the protein expression of MPST (Figure 4B).

Given that donor kinetics represent an important translational issue, the slow-releasing H_2_S donor GYY4137 was employed for parallel interventional experiments. Consistent with the findings for NaHS, treatment with GYY4137 effectively restored the expression levels of CSE and CBS (Figure 4C). Furthermore, hypoxia-induced upregulation of PCNA was significantly suppressed, accompanied by a marked reduction in phospho-ERK1/2 levels (Figure 4C). The above results indicated that H_2_S supplementation, utilizing either the fast-releasing donor NaHS or the slow-releasing donor GYY4137, reinstates the endogenous H_2_S-producing pathway and abrogates hypoxia-induced PASMC proliferation.

**Figure 5:**
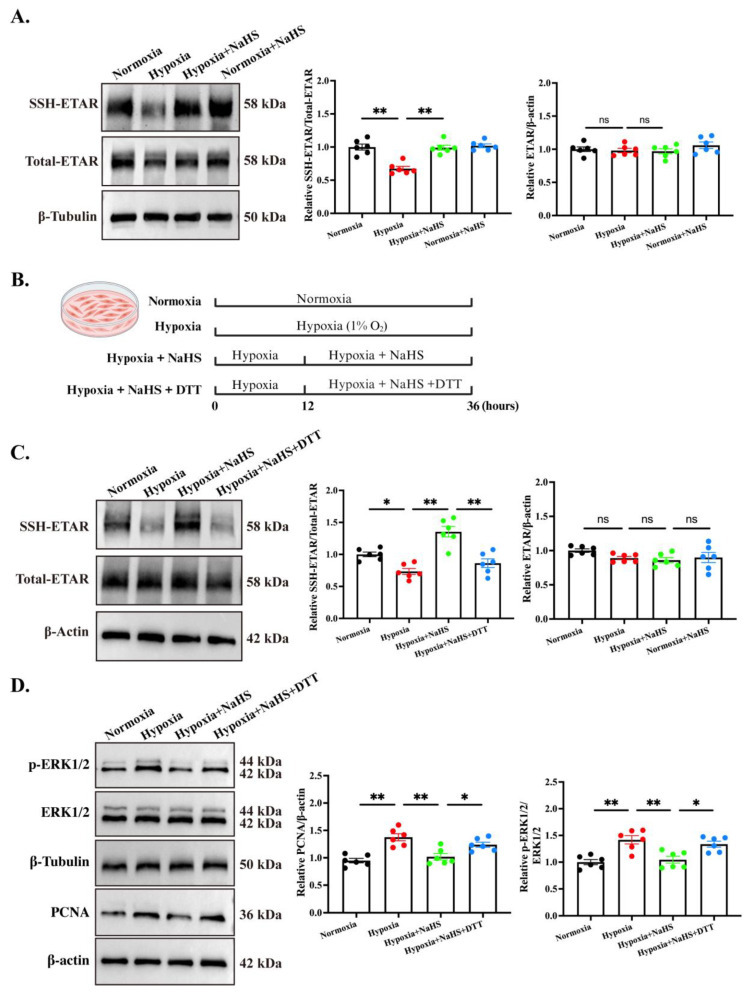
H_2_S inhibited hPASMC proliferation via upregulation of ETAR persulfidation. (**A**). Biotin-switch assay (BSA) quantitation of ETAR persulfidation and total ETAR in rat lung tissues. (**B**). Schematic diagram of cell grouping and treatment regimen. (**C**). BSA quantification of ETAR persulfidation and total ETAR in cultured hPASMCs. (**D**). Western blot detection of ERK1/2 phosphorylation and PCNA in hPASMCs. All data are expressed as mean ± SEM, *n* = 6. * *p* < 0.05, ** *p* < 0.01, ns: not statistically significant.

We previously showed that the increase in endogenous H_2_S production persulfidated ETAR and blocked ET-1-driven PASMC proliferation in vitro [19]. To test whether this post-translational switch operated in established HPH, we first quantified total ETAR protein in rat lung tissues. Neither hypoxia nor NaHS altered ETAR abundance (Figure 5A), excluding transcriptional or translational regulation. As we expected, hypoxia reduced ETAR persulfidation by 32.6%; NaHS restored it to normoxic levels (Figure 5A). To confirm causality, we further assessed ETAR persulfidation in hPASMCs. hPASMCs were exposed to 1% O_2_ for 12 h and then treated with NaHS for a further 24 h (Figure 5B). Hypoxia lowered ETAR persulfidation, increased phospho-ERK1/2, and raised PCNA (Figure 5C,D). NaHS reversed each change, whereas co-incubation with DTT abrogated protection (Figure 5C,D), proving that persulfidation is obligatory for the antiproliferative effect. Given the significance of persulfidation on ETAR, we additionally tested the *in vivo* effects of ET-1 on arterial pressure under control conditions, as well as in the presence of H_2_S. The results showed that intravenous administration of ET-1 (2 nmol/kg) induced a rapid elevation in systolic blood pressure in rats, peaking at 15–20 min with an average increase of 36.5 mmHg. Notably, pre-treatment with the H_2_S donor NaHS (56 μmol/kg) via intraperitoneal injection 30 min prior effectively antagonized the ET-1-induced pressor response. This intervention reduced the average blood pressure elevation to 13.0 mmHg, corresponding to an inhibition rate of 64.4%, which was deemed statistically significant (Figure S4).

Thus, late-initiated H_2_S therapy halts PASMC proliferation and vascular remodeling *in vivo* by re-establishing ETAR persulfidation and severing the ERK1/2 proliferative pathway.

## 4. Discussion

Based on a rat model of established hypoxic HPH, we demonstrated that late-initated H_2_S donor therapy reversed pre-existing hemodynamic dysfunction and vascular remodeling. Key observations were: (1) normalization of mPAP, RVSP and PAT/PET ratio; (2) a reduction in RVHI, indicating regression of right ventricular hypertrophy; (3) attenuation of medial thickening, luminal narrowing and muscularization in small pulmonary arteries; (4) suppression of PASMC proliferation, reflected by decreased PCNA and Ki-67 expression; (5) restoration of ETAR persulfidation, disrupting the ETAR/ERK1/2 proliferative pathway; and (6) across both cellular and animal levels, H_2_S donors were found to reverse the hypoxia-induced downregulation of key endogenous H_2_S-producing enzymes. The protective efficacy of H_2_S donors may stem from their ability to restore the expression of these enzymes, thereby reinstating the endogenous H_2_S biosynthetic machinery. Collectively, these data establish H_2_S as a HPH-reversing, rather than merely protective, agent in clinically manifest HPH.

The principal innovation of this study lies in the reconceptualization of H_2_S from a preventive mediator to a disease-reversing therapeutic agent. Earlier investigations, including our own, administered H_2_S concurrently with the injurious stimulus (hypoxia or monocrotaline) and documented prevention of disease initiation [16,17,18]. While these studies provided valuable mechanistic insights, their design diverged from clinical scenarios in which patients typically present with advanced, structurally entrenched pathology. To address this translational gap, we deferred H_2_S intervention until after three weeks of hypoxic exposure—a time point at which rats exhibited unequivocal elevations in mPAP, robust right ventricular pressure overload, and pronounced pulmonary vascular remodeling. Under these therapeutic intervention conditions, H_2_S rapidly reduced mPAP, normalized right ventricular afterload, and regressed medial hypertrophy and vascular muscularization. These findings provide compelling evidence that H_2_S can dismantle pre-existing vascular lesions despite persistent hypoxic stress. Therefore, these data establish that H_2_S is not merely required for baseline pulmonary vascular homeostasis; rather, repletion of the deficient H_2_S signal is sufficient to activate endogenous repair programs after maladaptive vascular structural remodeling has occurred. By shifting the intervention paradigm from prevention to reversal, this work provides a direct translational justification to date for targeting the H_2_S pathway in clinically manifest HPH.

The lethal progression of PAH is driven by relentlessly progressive vascular structural remodeling that culminates in right-heart failure; reversal of this process, therefore, represents a necessary condition of any curative strategy [33]. Excessive proliferation of PASMCs within the tunica media constitutes the dominant cellular driver of the vascular structural remodeling [34,35,36,37]. Our study demonstrated that therapeutic H_2_S administration significantly braked this engine, as evidenced by decreased Ki-67 and PCNA expression, regression of medial thickness, and reversal of small pulmonary artery muscularization. These cellular changes provided the mechanistic basis for the observed normalization of pulmonary artery pressure and right ventricular afterload. These antiproliferative effects were underpinned by the silencing of ERK1/2 signaling. The mitogen-activated protein kinase pathway, particularly ERK1/2, is a pivotal signaling pathway regulating cell proliferation [38,39]. Whereas earlier preventive protocols showed that concurrent H_2_S could blunt initial ERK1/2 phosphorylation [40], our therapeutic intervention model revealed that this signaling pathway remained pharmacologically targetable even in established, remodeled vasculature subjected to persistent hypoxia stress. H_2_S repletion not only halted further ERK1/2-driven mitogenesis but also actively reversed the established proliferative phenotype within the hypertensive pulmonary vasculature, thereby re-establishing physiologic balance between PASMC growth and apoptosis. To further validate the antiproliferative effect of H_2_S and address the potential influence of donor release kinetics, we conducted additional experiments using the slow-releasing H_2_S donor GYY4137. Consistent with our findings using NaHS, GYY4137 treatment restored the expression of CSE and CBS under hypoxic conditions, while significantly suppressing hypoxia-induced PCNA upregulation and phospho-ERK1/2. These results demonstrated that the inhibitory effect on hPASMC proliferation is not limited to fast-releasing H_2_S donors but is also reproduced by those offering sustained H_2_S release. This confirms that the observed biological effects are attributed to H_2_S itself, rather than donor kinetics, thereby strengthening the mechanistic evidence for H_2_S’s direct role in counteracting hypoxia-induced PASMC proliferation. By targeting the final common pathway of PASMC proliferation, H_2_S converted a previously considered irreversible structural defect into a reversible, and now reversed, phenotype.

The ET-1/ETAR axis is a canonical driver of PASMC proliferation [41,42]. In our previous study, we discovered that hypoxia might enhance the binding of ETAR with ET-1 and promote PASMC proliferation via a reduced ETAR persulfidation due to the deficiency of the endogenous H_2_S pathway [19]. In the present study, we elucidated the role of H_2_S-mediated persulfidation in the treatment of HPH, using both rat and PASMC models. Our data demonstrated that decreased endogenous H_2_S levels in HPH rat lungs were accompanied by significantly reduced ETAR persulfidation. Therapeutic administration of the H_2_S donor restored lung tissue H_2_S levels and markedly increased ETAR persulfidation. These molecular changes were accompanied by downstream improvements, including decreased ERK1/2 phosphorylation, reduced expression of PASMC proliferation markers, regression of pulmonary vascular structural remodeling, and decreased pulmonary artery pressure. Notably, these effects were blocked by DTT in hPASMCs, confirming the specificity of ETAR persulfidation-mediated mechanisms. Further, we examined the effects of ET-1 on arterial blood pressure *in vivo*. The presence of H_2_S effectively antagonized the increase in the blood pressure induced by ET-1, supporting the crucial role of H_2_S in endothelin receptor-mediated signaling and blood pressure regulation. Based on the abovementioned *in vivo* and *in vitro* findings, we proposed a model in which H_2_S exerted therapeutic effects on HPH through the upregulation of ETAR persulfidation, thereby inhibiting ETAR and its downstream ERK1/2 proliferative pathway. This nuanced mode of action may confer unique therapeutic advantages: effective suppression of pathological ET-1/ETAR signaling and PASMC proliferation while preserving the physiological functions of ET-1/ETBR in vascular tone regulation, potentially minimizing adverse effects associated with complete pathway blockade [43].

The present study focused on the persulfidation of ETAR as a key molecular mechanism of H_2_S therapy in HPH. However, it should be clarified that as a gaseous signaling molecule, H_2_S exerts biological effects through multiple targets and pathways, and its protective role may involve other unelucidated molecular targets and signaling pathways, warranting further investigation. Previous studies have demonstrated that H_2_S can induce vasodilation by enhancing the activity of ATP-sensitive potassium (K_ATP_) channels to promote the hyperpolarization of vascular smooth muscle cell membranes, thereby inhibiting calcium influx and reducing cell contractility [44]; or by enhancing the probability of channel opening through persulfidation [45]. Under hypoxic conditions, abnormal function of K_ATP_ channels in PASMCs easily induces cell depolarization and excessive calcium influx. H_2_S may restore their function to counteract this pathological process, ultimately improving vascular tone, which may represent one of the important mechanisms of H_2_S therapy for HPH. In addition, sodium–glucose cotransporters (SGLTs, especially SGLT2) have recently been implicated in the pathogenesis of cardiovascular and pulmonary vascular diseases, and their inhibitors have potential protective effects on pulmonary hypertension. There may be a crosstalk mechanism between H_2_S and SGLT signaling [46]. Both H_2_S and SGLT2 inhibitors can activate the PI3K/Akt/eNOS pathway to improve nitric oxide (NO) bioavailability and inhibit NADPH oxidase to reduce oxidative stress, thereby exerting vasodilatory and antiproliferative effects [47,48]; meanwhile, the antioxidant, anti-inflammatory and metabolic regulatory effects of H_2_S may further influence the SGLT-associated metabolic network and indirectly regulate pulmonary vascular function and structure [49]. Dysfunction of vascular endothelium is a core link in the development of HPH. As an endothelial protective molecule, H_2_S can promote NO production by enhancing eNOS activity, inhibit oxidative stress and inflammatory responses, and activate calcium-activated potassium channels in endothelial cells to induce endothelial cell membrane hyperpolarization and transmit electrical signals to smooth muscle cells, causing vasodilation [21] and inhibiting vascular remodeling and the progression of pulmonary hypertension. Notably, the direct effect of H_2_S on the heart itself may also play an important role in its therapy for HPH. Long-term HPH easily increases the pressure load on the right ventricle, leading to right ventricular hypertrophy and failure. H_2_S can directly act on cardiomyocytes to maintain myocardial homeostasis by inhibiting apoptosis, reducing oxidative stress, and improving mitochondrial function [50,51]. It can also regulate calcium homeostasis and inflammatory pathways to improve myocardial contractile function; simultaneously, by reducing pulmonary vascular resistance, it decreases the afterload of the right ventricle, and the dual benefits delay right ventricular structural remodeling and improve cardiac function [28]. In summary, H_2_S is involved in the treatment of HPH through the synergistic effect of multiple targets and pathways. Future molecular and functional experiments are needed to clarify the specific roles of each potential target so as to fully elucidate its mechanism and provide theoretical support for clinical translation.

Chronic hypoxia has been reported to cause mitochondrial dysfunction, exacerbate oxidative stress, and disrupt neuronal energy metabolism, ultimately leading to abnormalities in brain structure and function [52]; simultaneously, it triggers oxidative stress and neuroinflammation, accelerating neurodegenerative processes [53]. Furthermore, hypoxia activates oxygen sensors in the carotid body, which transmit signals to the medulla and stimulate neural pathways involving the nucleus tractus solitarius–rostral ventrolateral medulla–intermediolateral nucleus, enhancing sympathetic activity [54]. It can also activate peripheral chemoreceptors, reflexively stimulating the cardiovascular center and causing sustained sympathetic overactivation [55]. This not only exacerbates pulmonary hypertension but also increases systemic vascular resistance, induces systemic hypertension, and raises cardiac afterload. As an important endogenous gaseous signaling molecule, H_2_S was found to play an important protective role in the cardiovascular, nervous, and respiratory systems [56,57,58,59]. Therefore, under chronic hypoxic conditions, H_2_S donors might exert beneficial effects not only by improving pulmonary hypertension and inhibiting pulmonary vascular remodeling but also by mitigating systemic hypoxia-induced damage. Whether these systemic protective effects contribute to the overall improvement observed in the present study merits further investigation.

Although this study demonstrates the therapeutic potential of H_2_S using the classical rapid-release donor NaHS, clinical translation from preclinical models necessitates optimization of donor formulations to enhance therapeutic prospects. Future development might consider prioritizing the following three strategic avenues. First, pharmacokinetic optimization represents a critical priority. Preclinical evaluation of sustained-release and targeted H_2_S donors (e.g., GYY4137, AP39) might be advanced in more PAH models to achieve stable, sustained, and precise H_2_S delivery while minimizing potential side effects. Second, combination therapeutic strategies merit exploration. Given the pathophysiological complexity of PAH, monotherapy often yields limited efficacy. The pleiotropic mechanisms and favorable safety profile of H_2_S position it as a promising candidate for combination use with existing standard therapies (e.g., endothelin receptor antagonists, PDE5 inhibitors, and prostacyclin analogues). Rational cocktail approaches may yield superior therapeutic outcomes relative to single-agent regimens. Finally, comprehensive population-based studies are essential. Large-scale cohort investigations characterizing circulating H_2_S levels in patients with different PAH subtypes and severity grades may facilitate its validation as a biomarker for disease classification and precision medicine applications. Moreover, although putative mechanisms have been proposed, the precise dominant mechanisms underlying H_2_S-mediated reversal of PAH remain to be elucidated. System-level approaches that integrate proteomic and transcriptomic profiling analyses will facilitate the comprehensive characterization of the H_2_S-regulated molecular landscape, thereby enabling the identification of novel therapeutic targets and their cognate downstream effector pathways.

## 5. Conclusions

In summary, this study addresses the core question of whether H_2_S can reverse established HPH. Our findings demonstrate that therapeutic administration of an H_2_S donor effectively reverses hemodynamic abnormalities and pulmonary vascular structural remodeling in a well-established HPH model, with the mechanism closely linked to the inhibition of PASMC proliferation. This finding represents a pivotal shift for the role of H_2_S in HPH—from a preventative to a therapeutic agent. It provides robust experimental evidence and theoretical support for developing novel H_2_S-targeted therapeutic strategies for patients with clinical-stage HPH. Future research should focus on optimizing H_2_S donor formulations, refining treatment protocols, and exploring combination therapies with current clinical regimens to accelerate clinical translation.

## Data Availability

The original contributions presented in this study are included in the article/Supplementary Materials. Further inquiries can be directed to the corresponding author(s).

## Supplementary Materials

The following supporting information can be downloaded at: https://www.mdpi.com/article/10.3390/biomedicines14040760/s1, Figure S1: Pulmonary arterial hypertension model in rats induced by 3-week hypoxia exposure. Figure S2: Hypoxia exposure induced a downregulation of CSE protein expression in hPASMCs. Figure S3: NaHS treatment effectively reversed the hypoxia-induced downregulation of intracellular H_2_S levels. Figure S4: H_2_S inhibited ET-1-induced pressor response in rats.

## Abbreviations

The following abbreviations are used in this manuscript:

BSA	Biotin switch assay
CBS	Cystathionine-β-synthase
CSE	Cystathionine-γ-lyase
DTT	Dithiothreitol
ET-1	Endothelin-1
ETAR	Endothelin type A receptor
EVG	Elastic Van Gieson
FM	Fully muscularized
H_2_S	Hydrogen sulfide
hPASMCs	Human pulmonary artery smooth muscle cells
HPH	Hypoxia-induced pulmonary hypertension
LV	Left ventricle
mPAP	Mean pulmonary artery pressure
MPST	3-mercaptopyruvate sulfurtransferase
PAH	Pulmonary arterial hypertension
PASMC	Pulmonary artery smooth muscle cell
PAT	Pulmonary acceleration time
PET	Pulmonary ejection time
RMA	Relative media area
RMT	Relative media thickness
RV	Right ventricle
RVHI	Right ventricular hypertrophy index
RVSP	Right ventricular systolic pressure
SEM	Standard error of the mean
