# Supplementary material for: H2S Donor Therapy Reverses Established Pulmonary Arterial Hypertension and Pulmonary Vascular Structural Remodeling in Rats
Source: Biomedicines. 2026 Mar 26;14(4):760. doi: 10.3390/biomedicines14040760 (PMC13112977; doi:10.3390/biomedicines14040760)
Supplement: Supplementary file 1 [file biomedicines-14-00760-s001.zip › biomedicines-4152694-supplementary.pdf]

Supplementary Materials for

# $H_2S$ donor therapy reverses established pulmonary arterial hypertension and pulmonary vascular structural remodeling in rats

**Supplementary Methods:**

***1. Fluorescent Probe Detection of Intracellular $H_2S$ Levels***

In hPASMCs that had been treated as required, the culture medium was removed, and the cells were gently washed twice with PBS to eliminate residual serum and potential interfering substances. Subsequently, the cells were incubated with the $H_2S$ fluorescent probe working solution at 37 °C for 30 min in the dark. After incubation, the staining solution was discarded, and the cells were gently washed 2–3 times with PBS to remove unbound probe. The cells were then fixed with 4% paraformaldehyde, followed by nuclear counterstaining with DAPI. Finally, fluorescence images were acquired using a confocal laser scanning microscope under light-protected conditions.

***2. Measurement of Carotid Arterial Pressure in Rats***

Male Sprague-Dawley (SD) rats (180-220 g) were purchased from Charles River Biotechnology Co., Ltd (Beijing, China). The rats were anesthetized with 20% urethan (10 mL/kg, intraperitoneally), and tracheal intubation was performed to facilitate mechanical ventilation. For hemodynamic monitoring, the right carotid artery was cannulated using a catheter of suitable diameter. The catheter was connected to a pressure transducer, which was linked to a biosignal acquisition system for real-time monitoring and recording of arterial pressure. Rats were randomly divided into three groups (n= 6 per group, total 18 rats): (1) Control group: Vehicle treatment; (2) ET-1 group: Intravenous injection of ET-1 at a dose of 2 nmol/kg; (3) ET-1+NaHS group: Intraperitoneal injection of NaHS (56μmol/kg) followed 30 min later by intravenous ET-1 (2 nmol/kg). After ET-1 injection, blood pressure was continuously recorded for a minimum of 30 min. The inhibitory percentage of NaHS on ET-1-induced pressor response was calculated as follows: inhibitory percentage (%) = [(blood pressure increase in ET-1 group) - (blood pressure increase in ET-1+NaHS

group)] / (blood pressure increase in ET-1 group) × 100%.

**Supplementary Data:**

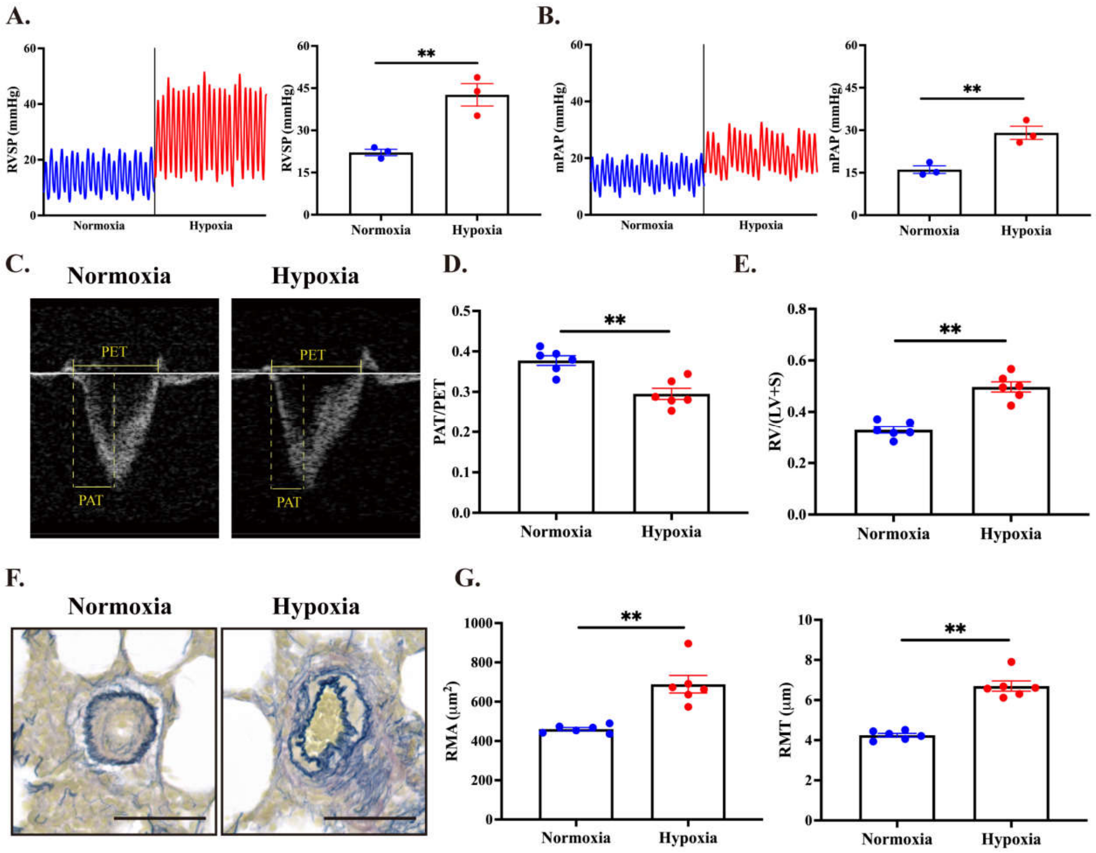


**Figure S1**. Pulmonary arterial hypertension model in rats induced by 3-week hypoxia exposure. A. Right ventricular systolic pressure measured by right heart catheterization. B. Mean pulmonary artery pressure measured by right heart catheterization. C. Representative echocardiographic images of pulmonary arterial blood flow spectra. D. Statistical analysis of the echocardiographic index PAT/PET ratio. E. Statistical analysis of right ventricular hypertrophy index. F. Representative image of rat lung tissue stained with EVG, scale bar = 50μm. G. Statistical analysis of relative media area and relative media thickness of small pulmonary arteries. All data are presented as mean ± SEM; n = 3–6. $^{**}p < 0.01$.

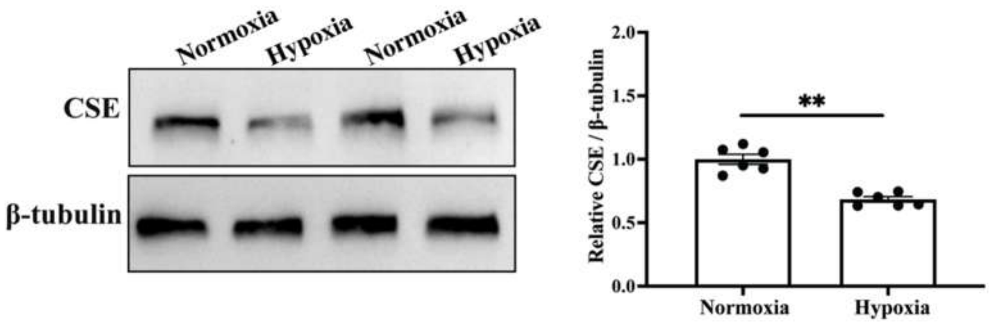


**Figure S2**: Hypoxia exposure induced a downregulation of CSE protein expression in hPASMCs. CSE protein expression in hPASMCs after 12 h of hypoxia was analyzed via immunoblotting. All data are expressed as mean ± SEM, n=6. $^{**}p<0.01$.

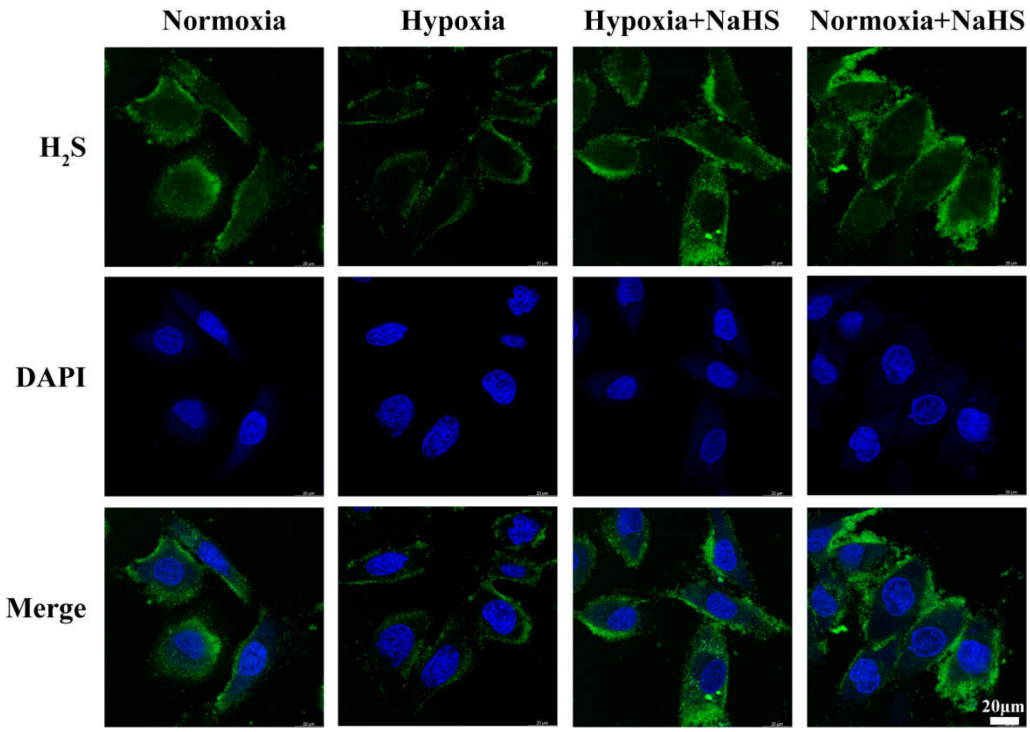


**Figure S3**: NaHS treatment effectively reversed the hypoxia-induced downregulation of intracellular $H_2S$ levels. $H_2S$ levels in hPASMCs treated with 100 μM NaHS for 24 hours were detected using a fluorescent probe assay. $H_2S$ (green) and DAPI (blue); scale bar = 20 μm.

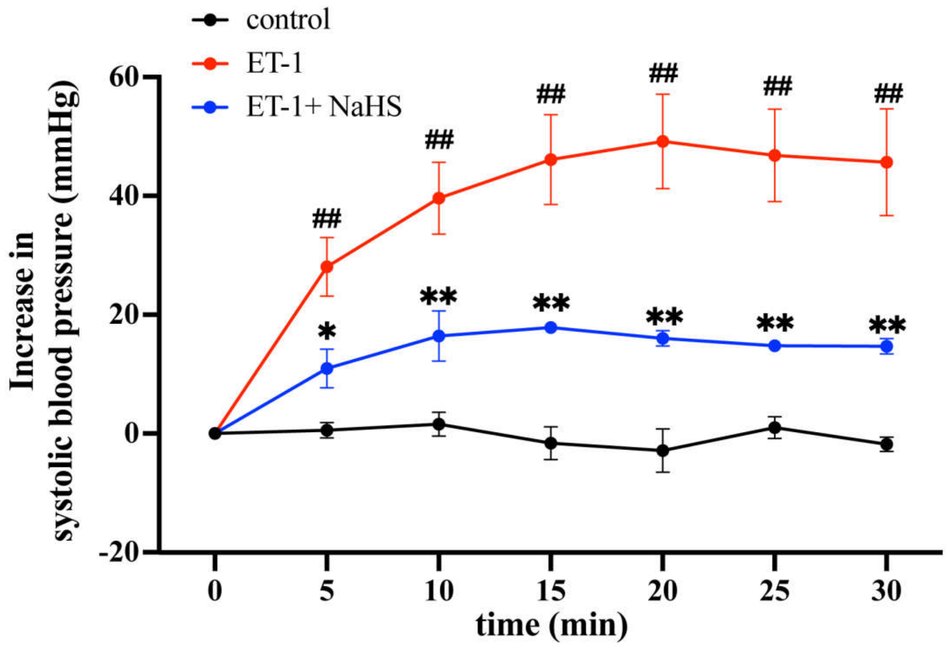


**Figure S4**: $H_2S$ inhibited ET-1-induced pressor response in rats. Systolic blood pressure was monitored via right carotid artery catheterization. ## vs. control group, $p<0.01$; * vs. ET-1 group, *$p<0.05$, **$p<0.01$.
